# Sensitivity of three commercial tests for SARS-CoV-2 serology in children: an Italian multicentre prospective study

**DOI:** 10.1186/s13052-022-01381-9

**Published:** 2022-12-02

**Authors:** Elisabetta Venturini, Sabrina Giometto, Agnese Tamborino, Laura Becciolini, Samantha Bosis, Giovanni Corsello, Paolo Del Barba, Silvia Garazzino, Andrea Lo Vecchio, Alessandra Pugi, Sara Signa, Giacomo Stera, Sandra Trapani, Guido Castelli Gattinara, Ersilia Lucenteforte, Luisa Galli

**Affiliations:** 1 Infectious Disease Unit, Meyer Children’s Hospital, Florence, Italy; 2 Unit of Medical Statistics, Department of Clinical and Experimental Medicine, University of Pisa, Pisa, Italy; 3 Clinical Chemistry and Microbiology Laboratory, Meyer Children’s Hospital, Florence, Italy; 4 Pediatric Highly Intensive Care Unit, Fondazione IRCCS Ca’ Granda Ospedale Maggiore Policlinico, Milan, Italy; 5 Unit of Pediatrics and Neonatal Intensive Therapy, Department of Promotion of Maternal and Infantile and Internal Medicine Health, and Specialist Excellence “G. D’Alessandro”, University of Palermo, Palermo, Italy; 6 Department of Pediatrics, IRCCS San Raffaele Scientific Institute, Milan, Italy; 7 Paediatric Infectious Diseases Unit, Regina Margherita Children’s Hospital, University of Turin, Turin, Italy; 8 Section of Paediatrics, Department of Translational Medical Sciences, University of Naples Federico II, Naples, Italy; 9 Clinical Trial Office, Meyer Children’s Hospital, Florence, Italy; 10 Infectious Diseases Unit, IRCCS Istituto Giannina Gaslini, Genoa, Italy; 11 Postgraduate School of Pediatrics, University of Bologna, Bologna, Italy; 12 Department of Health Sciences, University of Florence, Florence, Italy; 13 Vaccination Unit, University Hospital Paediatric Department, Bambino Gesù Children’s Hospital, IRCCS, Rome, Italy

**Keywords:** COVID-19, SARS-CoV-2, Electrochemiluminescent immunoassay (ECLIA), Enzyme-linked immunosorbent assay (ELISA), Children

## Abstract

**Background:**

US Food and Drug Administration has issued Emergency Use Authorizations for hundreds of serological assays to support Severe Acute Respiratory Syndrome Coronavirus 2 (SARS-CoV-2) diagnosis. The aim of this study is to evaluate, for the first time in children, the performance of three widely utilized SARS-CoV-2 serology commercial assays, Diesse Diagnostics (IgG, IgA, IgM) and Roche Diagnostics, both Roche Nucleocapsid (N) IgG and Roche Spike (S) IgG assays.

**Methods:**

Sensitivity and 95% confidence intervals (CIs) were estimated for each of the three different serological tests and mixed and direct comparison were performed.

Univariate and multivariate Poisson regression models were fitted to calculate incidence rate ratios and 95% CIs as estimate of the effects of age, gender, time on the serology title. A *p*-value < 0.05 indicated statistical significance.

**Results:**

Overall, 149 children were enrolled in the study. A low sensitivity was found for Diesse IgA, IgM and IgG. Compare to Diesse, Roche S had a higher sensitivity at 15–28 days from infection (0.94, 95%CI: 0.73–1.0) and Roche N at 28–84 days (0.78, 95%CI: 0.58–0.91). When a direct comparison of IgG tests sensitivity was feasible for patients with pairwise information, Roche S and Roche N showed a statistically significant higher sensitivity compared to Diesse in all the study periods, whereas there was no difference between the two Roche tests.

**Conclusion:**

Roche S and Roche N serology tests seem to better perform in children. Large prospective studies are needed to better define the characteristics of those tests.

**Supplementary Information:**

The online version contains supplementary material available at 10.1186/s13052-022-01381-9.

## Introduction

The Severe Acute Respiratory Syndrome Coronavirus 2 (SARS-CoV-2) was declared a global pandemic in March 2020 by the World Health Organization (WHO) [1, 2].

At present, the standard diagnostic confirmatory test for Coronavirus Disease (COVID)-19 is based on the detection of nucleic acids of SARS-CoV-2 by nucleic acid amplification tests on respiratory samples [1]. Antigen tests and rapid molecular-based tests are an alternative, being suitable for use as point of care [3].

Despite the limited role in the diagnosis of acute infection, serological tests are important for surveillance purposes and epidemiological assessment of the immunization status of the population [4]. Moreover, serology is a cornerstone in the definition of cases of Multisystem Inflammatory Syndrome in Children (MIS-C), when the clinical presentation is suspected. Another possible use is the assessment of COVID-19 vaccine immune responses and durability [4, 5].

Although the humoral response to SARS-CoV-2 currently is incompletely defined, it appears that approximately 60% of infected individuals produce IgM antibodies about 4 days post-symptoms onset, with a peak between 14 and 21 days and then decline. IgG levels begin to rise at about 7–14 days, peaking at around day 25 [6]. It is unclear how long IgG levels are sustained although some individuals has detectable IgG antibodies at least 6–7 months after onset [7, 8]. Moreover, the evaluation of IgA levels in a larger number of COVID-19 patients is still lacking [9, 10].

The US Food and Drug Administration (FDA) has issued Emergency Use Authorizations for hundreds of serological assays to support COVID-19 diagnosis, and at present more than 1 thousand immunoassays are either commercially available or in development [11, 12]. According to the WHO indication, ≥ 95% and ≥ 97% as acceptable criteria for sensitivity and specificity, respectively [13]. Test types include formal laboratory-based assays such as enzyme-linked immunosorbent assays (ELISA), chemiluminescent immunoassays (CLIA) and point-of-care rapid lateral flow immunoassays (LFIA) [1, 4]. Despite the large number of available serology kits, studies on serological tests in children with SARS-CoV-2 infection are limited.

The main objective of this study was to evaluate the sensitivity of three widely utilized SARS CoV-2 serology commercial assays, Diesse Diagnostics (IgG, IgA, IgM) and Roche Diagnostics, both Roche N (IgG) and Roche S (IgG) assays. The secondary aim was to perform a head-to-head comparison of the diagnostic sensitivity of those three assays.

## Materials and methods

### Study population

The present study was promoted by the Italian Society of Pediatric Infectious Diseases, as part of a prospective study on the epidemiology and clinical characteristics of pediatric COVID-19 [14].

Overall, seven pediatric hospitals across Italy (Milan, Genoa, Bologna, Naples, Florence, Turin, Palermo) participated to this study. The Infectious Diseases Unit at Meyer Children’s University Hospital, Florence was the coordinator centre of this study.

Each child (aged < 18 years) with a diagnosis of acute SARS-CoV-2 infection or MIS-C during the study period (1st March 2020 - 30th June 2021) was screened for inclusion. All cases were considered confirmed in case of viral RNA detection from oropharyngeal or nasopharyngeal swab sample by real-time polymerase chain reaction (rtPCR), used as reference standard for SARS-CoV-2 diagnoses, performed at Immunology and Molecular Microbiology Unit, Meyer Children’s University Hospital, Florence, Italy using standardized techniques and according to manufacturers’ instructions.

Only symptomatic children were included in the analysis.

For each child enrolled an additional serum sample (0.5 mL) was obtained on the occasion of venipuncture for the study tests. All serum samples were centralized at Meyer Children University Hospital, in Florence, transported on dry ice and then stored at − 20 °C on arrival. These samples were then thawed and centrifuged before performing the assays.

Timing of serology was categorized according to the number of days following symptoms onset in three-time intervals (0–14, 15–28 and 29–84 days).

Demographic and clinical data were collected for each child and recorded into the study database. A single researcher for each institution collected and deidentified clinical data by using an electronic clinical registration form. All data were subsequently merged into a single database specifically designed for statistical analysis.

This study was approved by the Pediatric Ethics Committee of the Tuscany Region, Florence, Italy (PED-COVID-19, approved on 17th March 2020). This study was undertaken in accordance with good clinical practice guidelines and the Declaration of Helsinki. Written informed consent was obtained from parents/caregivers, and the patient if appropriate. This manuscript follows the rules of the STARD checklist for.

reporting of studies of diagnostic accuracy (Supplementary material, Table S1).

### SARS-CoV-2 serology kit

Serology tests were performed at the Clinical Chemistry and Microbiology Laboratory, Meyer Children’s University Hospital, Florence, Italy using standardized techniques and according to manufacturers’ instructions.

Anti-SARS-CoV-2 antibodies were assessed by using three commercially available immunoassays: (1) Enzy-Well SARS-CoV-2 IgM, IgG, IgA (Diesse Diagnostics, Siena, Italy), referred in this paper as Diesse (2) Elecsys Anti-SARS-CoV-2 N (anti-N IgG) on the Cobas e801 and Cobas e402 analyzers (Roche Diagnostics, Switzerland), referred as Roche N and (3) Elecsys Anti-SARS-CoV-2 S (anti-S IgG) on the Cobas e801 and Cobas e402 analyzers, referred as Roche S.

Diesse is based on the ELISA technique, whereas Roche N and Roche S are an ElectroChemiLuminescence ImmunoAssay (ECLIA) tests.

Assays results were reported as numeric values in the form of an index (signal sample/signal calibrator), interpreted as qualitative results according to the manufacturers’ cut-off for Roche N and for Diesse assays and as concentration (U/mL) for the quantitative Roche S assay (Table 1).

**Table 1:** The four serological assays used in this study

Assay	Manufacturer	Method	Antibody	Antigen	Cut-off
Enzy-Well SARS-CoV-2	Diesse	ELISA	IgA, IgM, IgG	S, N, E, M	> 1.1 (index)
Elecsys Anti-SARS-CoV-2 N	Roche	ECLIA	IgG	N	neg/pos
Elecsys Anti-SARS-CoV-2 S	Roche	ECLIA	IgG	S	> 0.4 U/mL

Legends: *S* Spike, *N* Nucleocapsid, *E* Envelope, *M* Membrane

The readers of the index tests and reference standard were not blind to the results of the other tests, as the results of those tests are objective.

### Statistical analysis

Continuous variables were reported as mean and standard deviations (SDs), or median and interquartile ranges (IQRs), according to their distribution. Categorical variables were expressed as frequencies and percentages.

Sensitivity and 95% confidence intervals (CIs) were estimated for each of the three serological tests and for the Diesse assay they were estimated for each class of immunoglobulins separately. Mixed and direct comparison were performed on all of the subjects and on only those with pairwise information on both serological tests, respectively. Differences of sensitivity between the three serological tests were evaluated using Chi-square and Mcnemar test as appropriate.

Univariate and multivariate Poisson regression models with robust variance were fitted to calculate incidence rate ratios (IRR) and 95% CIs as estimate of the effects of the explanatory variables (age, gender, time) on the response variable (serology). A *p*-value < 0.05 indicated statistical significance. Statistical analysis was performed using R statistic, version 4.1.0.

## Results

### Study population

Overall, 149 children with a diagnosis of acute symptomatic SARS-CoV-2 infection (131/149, 87.9%) or MIS-C (18/149, 12.1%) during the study period were included in the present study. The majority of the patients were males (55%), of Caucasian origin (68.5%), and their median age was 58.4 (IQR 4.7–148.45) months. Twelve patients (8.1%) were neonates. All patients were not vaccinated for SARS-CoV-2 infection. Overall, 97.3% of participants were hospitalized. Five out of 149 patients (3.4%) required invasive ventilation support and no deaths were reported.

A pre-existing condition was present in 34 (22.8%) children and 17 (11.4%) had a history of premature birth. The most common associated diseases were neurological and metabolic disorders (8/149, 5.3%) (Table 2). Fever was the most common sign (79.9%), followed by cough (22.8%) and rhinitis (19.5%). Co-infections were searched in a minority of patients (11.4%) and mainly found in the respiratory and urinary tract.

**Table 2:** Characteristics of the study population

Characteristic	N of cases (%), *N* = 149
Gender	
Male	82 (55)
Female	67 (45)
Racial or ethnic group	
Caucasian	102 (68.5)
American	13 (8.7)
African	4 (2.7)
Asian	4 (2.7)
Other/non reported	26 (17.4)
Underlying chronic diseases	
Total	34 (22.8)
Neurological and metabolic disorders	8 (5.3)
Complex genetic syndromes	5 (3.3)
Cancers	5 (3.3)
Obesity	5 (3.3)
Kidney diseases	2 (1.3)
Cardiovascular diseases	2 (1.3)
Endocrine disorders	2 (1.3)
Asthma	2 (1.3)
Hematologic diseases	1 (0.6)
Rheumatologic diseases	1 (0.6)
Immunodeficiency	1 (0.6)
Presenting signs/symptoms	
Fever	119 (79.9)
Cough	34 (22.8)
Rhinitis	29 (19.5)
Dyspnoea	29 (19.5)
Diarrhoea	26 (17.4)
Vomit	23 (15.4)
Pharyngodynia/pharyngitis	23 (15.4)
Skin rash	19 (12.8)
Abdominal pain	16 (10.7)
Conjunctivitis	13 (8.7)
Smell and taste alterations	10 (6.7)
Seizures	5 (3.4)
Chest pain	5 (3.4)
Arthralgia	3 (2)
Complications	
Pneumonia	32 (21.5)
Severe acute respiratory illness	13(8.7)
Acute respiratory distress syndrome	4 (2.7)

Treatments for SARS-CoV-2 infection were prescribed in 29.5% of patients. The most frequently used drugs were systemic steroids (20.8%), followed by macrolides (7.4%), hydroxychloroquine (6%), monoclonal antibodies (4.7%) and remdesivir (4%).

### Serological test

In our population, the median delay between symptoms onset and serology testing was 7 days (IQR 3–21 days). No adverse events were reported from performing venipuncture.

**Fig. 1:**
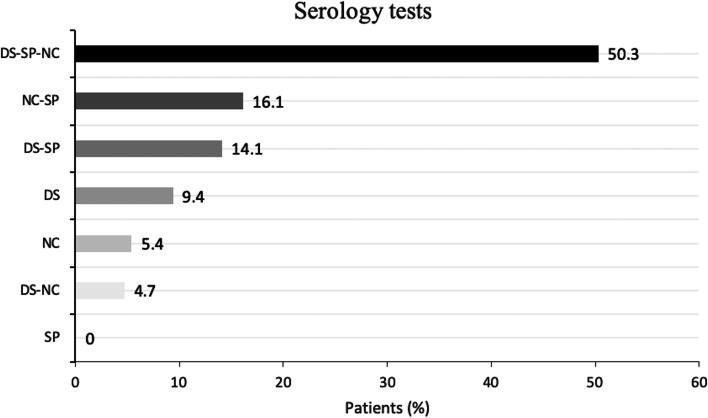
Distribution of serology tests according to patients. (SP: Roche S; NC: Roche N; DS: Diesse)

All the three serological tests were performed in 50.3% of patients while two tests were, respectively, performed in 16.1% (Roche N and Roche S), 14.1% (Roche S and Diesse) and 4.7% (Roche N and Diesse) cases (Fig. 1).

The sensitivity of the three serological tests was calculated for each time interval (0–14, 15–28 and 29–84 days) and reported in Table 3. A low sensitivity was found for Diesse IgA in all the intervals, with a higher value between 15 and 28 days (0.44, 95%CI: 0.22–0.69). Similarly, Diesse IgM performed better in the same time interval (0.67, 95%CI: 0.41–0.87). The lower sensitivity was found for Diesse IgG in all the time intervals.

**Table 3:** Sensitivity and 95% CI stratified by time for Diesse test, Roche S, Roche N

Test	0–14 days				15–28 days				28–84 days			
	Positive, N (*N* = 104)	Sensitivity (95% CI)	*p*-value	*p*-value	Positive, N (*N* = 18)	Sensitivity (95% CI)	*p*-value	*p*-value	Positive, N (*N* = 27)	Sensitivity (95% CI)	*p*-value	*p*-value
Diesse IgA	15	0.14 (0.08–0.23)			8	0.44 (0.22–0.69)			4	0.15 (0.04–0.34)		
Diesse IgM	15	0.14 (0.08–0.23)			12	0.67 (0.41–0.87)			16	0.59 (0.39–0.78)		
Diesse IgG	19	0.18 (0.11–0.27)	Ref	–	5	0.28 (0.1–0.53)	Ref	–	1	0.04 (0–0.19)	Ref	–
Roche S	43	0.41 (0.32–0.51)	**<0.001**	Ref	17	0.94 (0.73–1.0)	**<0.001**	Ref	17	0.63 (0.42–0.81)	**<0.001**	Ref
Roche N	40	0.38 (0.29–0.49)	**0.002**	0.78	12	0.67 (0.41–0.87)	**0.05**	0.09	21	0.78 (0.58–0.91)	**<0.001**	0.37

Legends: Ref = reference test

The sensitivity of Roche S was the highest, reaching 0.94 (95%CI: 0.73–1) at 15–28 days. Roche N was the test with a higher sensitivity in the last time interval (29–84 days).

Thereafter, the performance of SARS-CoV2 IgG of the three commercially available tests was compared (Table 3). In particular, the sensitivity of Roche S and Roche N were significantly higher compared to Diesse in all the three timeframes (*p* <  0.001 for all the tree intervals; *p* = 0.002, *p* = 0.05 and p <  0.001, respectively). Roche S and Roche N did not differ regarding sensitivity in all the study periods (*p* = 0.78, *p* = 0.09, *p* = 0.37, respectively).

In addition, direct comparison of sensitivity and 95%CI between IgG tests in those patients with pairwise information (Supplementary material, Table S2) showed consistent results with the ones encountered in direct comparison of three commercially available tests (Table 3).

Finally, sensitivity and 95%CI of combination of tests was calculated. However, the combination of tests did not significantly increase the sensitivity compared to the use of a single test, excepting for the comparison with Diesse IgG (Table 4). Moreover, any of the combinations was statistically superior to the others (Supplementary material, Table S3).

**Table 4:** Sensitivity and 95%CI between combination of tests in subjects with information on Diesse, Roche S and Roche N

Test	0–14 days		15–28 days		29–84 days	
	Positive, N (*N* = 46)	Sensitivity (95%CI)	Positive, N (*N* = 11)	Sensitivity (95%CI)	Positive, N (*N* = 18)	Sensitivity (95%CI)
Diesse IgG + Roche S	27	0.59 (0.43–0.73)	11	1 (0.72–1.00)	15	0.83 (0.59–0.96)
Diesse IgG+Roche N	26	0.57 (0.41–0.71)	10	0.91 (0.59–1.00)	15	0.83 (0.59–0.96)
Roche S+Roche N	28	0.61 (0.45–0.75)	11	1 (0.72–1.00)	15	0.83 (0.59–0.96)

**Fig. 2:**
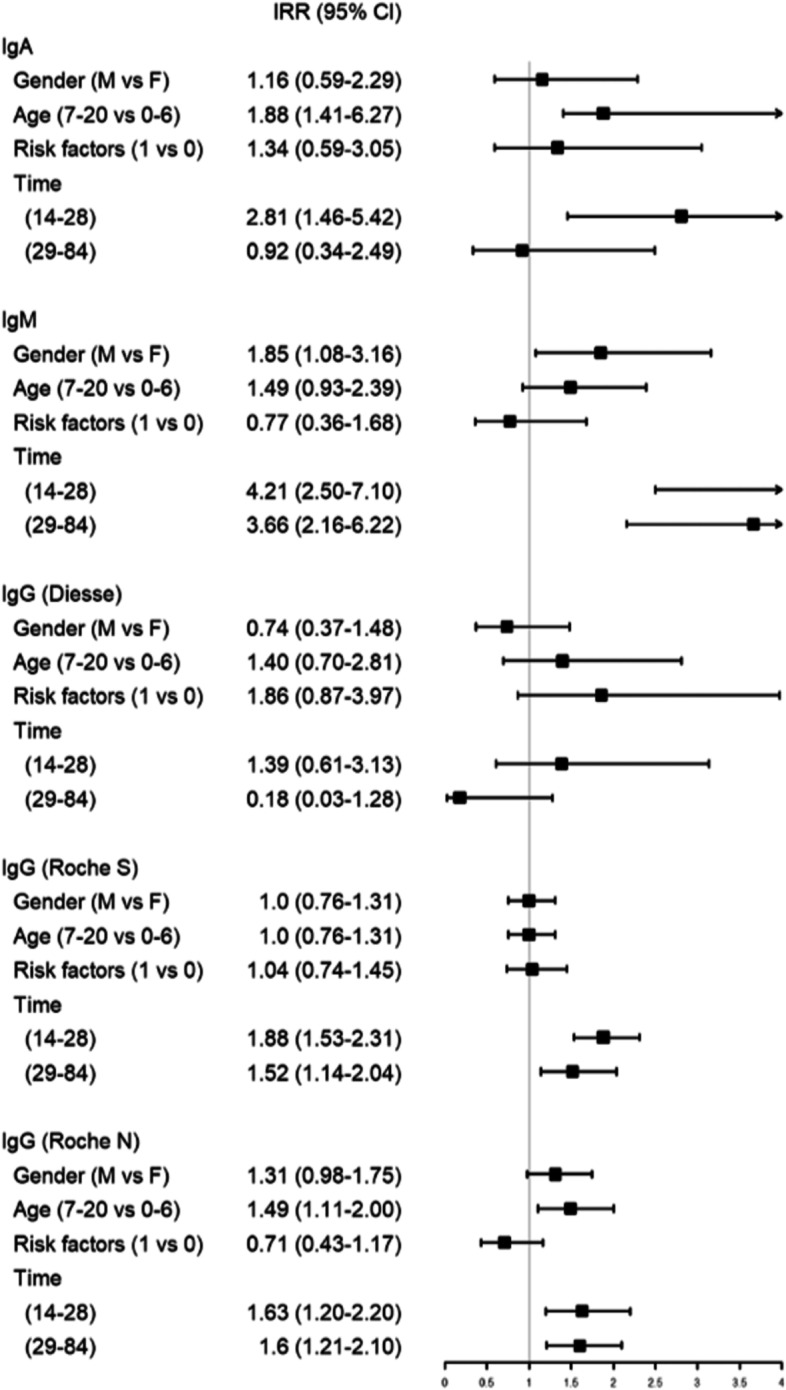
Forest plot of Poisson univariate regression models with robust variance to estimate the effect of gender, age, risk factors and time (independent variables) on the serology (dependent variable). IRR of time intervals was calculated using 0–14 days

Poisson univariate regression models showed that Diesse IgA sensitivity was significantly associated with age older (7–20 years vs 0–6 years) and time interval 15–28 days, Diesse IgM to gender and time intervals (14–28 days and 29–84 days), whereas Diesse IgG had no significant association with age, gender, time intervals and risk factors. Roche S and Roche N were significantly associated with time intervals 14–28 and 29–84, and Roche N also with age older (7–20 vs 0–6 years) (Fig. 2).

According to multivariate analysis, IgA sensitivity was still significantly influenced by time interval 14–28 days, adjusted for age (IRR: 2.33, 95%CI 1.12–4.83) and IgM sensitivity remains significantly influenced by time intervals 14–28 and 29–84 days, adjusted for gender (14–28 days: IRR 3.95, 95%CI 2.38–6.55; 29–84 days: IRR 3.46, 95%CI 2.04–5.85). Finally, IgG and Roche N remains significantly influenced by time intervals 14–28 and 29–84 days, adjusted for age (14–28: 1.49, 95%CI 1.09–2.04; 29–84: 1.51, 95%CI 1.15–1.99).

## Discussion

This multicentre prospective study describes the test performance of three anti-SARS-CoV-2 assays on 149 children with rRT-PCR confirmed SARS-CoV2 infection. To the best of our knowledge, this is the first study comparing the performance of three widely utilized SARS-CoV-2 serology commercial assays in children. The main finding of our study is the high sensitivity of CLIA assays (Roche S and Roche N) after 14 days since symptoms onset. Moreover, CLIA tests (Roche N and Roche S) were found to be significantly more sensitive than ELISA test (Diesse). In fact, a low sensitivity was found for Diesse IgA, IgM and IgG in our study, and the lowest sensitivity was found for Diesse IgG in all the time frames.

Few studies are available on SARS-CoV-2-specific IgA [9, 10]. The addition of IgA to serological tests analyzing IgM and IgG could increase the sensitivity of SARS-CoV-2 diagnosis in the early stage of infection [15]. In fact, the involvement of the upper airways, highly containing mucosal immune cells, could explain IgA production. In a study by Chiereghin et al. the sensitivity of ELISA IgA was 0.84 and significantly improved overtime in symptomatic patients, resulting in an overall sensitivity of 0.94 [16]. On the contrary, according to our results, Coste et al. demonstrated an insufficient performances of IgA and IgM for the diagnosis of COVID-19 [17]. Furthermore, some studies reported a cross-reactivity of ELISA IgA with other respiratory viruses such as influenza A and B and with other human coronaviruses [18–20].

In our study, IgM and IgA detected by ELISA performed better in the time interval 15–28 days from symptom onset while, Diesse IgG had a low sensitivity in all the time intervals. In general, the sensitivity of IgG and IgM based tests was low in the first week (1–7 days) of symptom onset and high in the third week or later (> 14 days) [1]. A metanalysis by Vengesai et al. showed higher pooled sensitivity with IgG and IgM based ELISA tests of 0.83 and 0.84, respectively [1]. Similarly, other three meta-analysis observed similar pooled sensitivity ranging from 0.70 (95% CI 0.55–0.84) to 0.84 (95% CI 0.70–0.92) [21–23]. The lower sensitivity of ELISA IgG found in this study compared to literature could be related to the small number of patients tested after 14 days since symptoms onset [1, 24, 25]. Moreover, the difference between literature data and our results on ELISA sensitivity could have few other explanations. In fact, it could be also be attributed to the targeted SARS-CoV-2 antigens contained in each kit and to the ELISA commercial kit used, considering also that the cut-off might also play a role [19]. Another important difference is that the available literature is limited to the adult population. The dynamics of the antibody response has been well described in adults [26], while there are few data in the pediatric population. A difference in the distribution, maturation and functioning of viral receptors has been mentioned as a possible reason for the age-related peculiarities [27]. In a in a Spanish multicenter study on 324 SARS-CoV-2 rRT-PCR positive children, 24% of them failed to seroconvert after the infection and patients with mild disease and shorter time to rRT-PCR negativity seroconverted less often than patients with more severe disease and who had more prolonged rRT-PCR positivity [26]. Moreover, studies of MIS-C suggested that not all patients developed detectable antibodies despite a strong dysregulated immune response [28, 29]. The evaluation of tests sensibility concerning the disease severity and special conditions such as MIS-C was not performed in our study, due to the limited number of cases.

Overall, CLIA tests exhibited a better sensitivity in our study population, with a better performance of Roche S in the interval 15–28 days. The sensitivity of Roche S and Roche N has been also evaluated in another study on adults, showing a sensitivity of 0.96 (95% CI 0.92–0.98) and 0.92% (95% CI 0.87–0.96), respectively [30].

We found that Roche S had a higher sensitivity at 15–28 days and Roche N at 28–84 days. This trend toward higher sensitivity over time is in accordance with previous studies [31].

The results in the literature are controversial. In a French study on 68 patients between 7 and 81 years of age eight commercial assays based on CLIA, ELISA and enzyme-linked fluorescent assay (ELFA) technologies were compared [32]. In this study, Wantai ELISA showed the best sensitivity, whereas Liaison CLIA the worst one [32]. In another study by Wolff et al., the highest overall sensitivity among the examined methods (Roche N CLIA, Liaison CLIA and Euroimmun ELISA) was achieved by using Euroimmun ELISA with a combined detection of IgG/IgA (0.86, 95%CI 0.78–0.92) [33].

In contrast, some studies demonstrated the superior performance of CLIA-based technique over ELISA-based ones, similarly to our study [34, 35]. In a study by Schnurra and colleagues comparing seven commercial antibody tests including Euroimmun ELISA, Siemens CLIA and Roche CLIA, the highest sensitivity was obtained by Siemens antibody testing followed by Roche and Euroimmun [34]. Other studies revealed that the diagnostic performance of CLIA tests is comparable to ELISA [10, 36–40]. In an Italian study on 184 serum samples from 130 COVID-19 patients and 54 SARS-CoV-2 negative subjects, four CLIA assays (Abbott SARS-Cov2 IgG, Roche N, Ortho SARS-CoV-2 total and IgG) and one ELISA (Diesse ENZY-WELL SARS-CoV-2 IgG) assay were compared [40]. The overall sensitivity of Roche N CLIA and Diesse ELISA was 0.78 (95% CI 0.70–0.85) and 0.83 (95% CI 0.75–0.98), respectively. A higher sensitivity was reached after 12 days since symptom onset. Another study by the same authors suggested that IgG levels measured by Maglumi CLIA and Euroimmun ELISA assays were comparable and the clinical agreement between these methods was 0.90 [10]. In another study by Egger et al., SARS-CoV-2 antibodies were measured with the Elecsys assay (Roche N CLIA) and the Edi ELISA in 64 patients, showing a sensitivity of 1.00 for Roche and of 0.94 for Edi ELISA 15–22 days after symptom onset [38].

Some studies suggested that combining N- and S-based tests may enhance true positivity and can be beneficial when extremely sensitive antibody tests are not accessible [30, 34]. However, in our study, the combination of tests did not significantly increase the sensitivity compared to the use of a single test (except for Diesse), nor did a combination performed better than the others. Similarly, in the study by Andrey et al., either Roche S alone or Roche S and Roche N parallel testing (either one positive leading to a positive result) displayed a sensitivity of 100% [30].

Moreover, a secure benefit of combining serology using both anti-N and anti-S antibody detection would be the capability of differentiating antibodies induced by SARS-CoV-2 infection (with both anti-S and anti-N antibodies) versus vaccine-induced antibodies (only anti-S antibodies) [30]. This application would be more useful in view of the recent authorization of anti-SARS-CoV-2 vaccines also for the pediatric age [41, 42].

The main limitation of our study is the small number of children enrolled. Moreover, the three assays were not available for all the enrolled patients due to the insufficient blood samples obtained in some cases, especially in young children. Another limitation is that the serology was not done at standardized timing but on the occasion of blood tests performed during hospitalization. This point led to the fact that in our study the median timing of serology was of 7 days, whereas the best sensitivity was reached after 14 days since symptoms onset. Therefore, data regarding serology after 14 days since symptoms onset were limited to a few patients. Finally, data on serology at follow-up were not available in this study.

## Conclusions

Serological tests could play a role in the diagnosis and follow-up of children with SARS-CoV-2 infection, and CLIA tests seem to better perform in this population. Large prospective studies are needed to better define the characteristics of those tests in the pediatric population.

## Supplementary Information

**Additional file 1:** **Table S1.** STARD checklist for reporting of studies of diagnostic accuracy.

**Additional file 2:** **Table S2.** Direct comparison of sensitivity and 95%CI between tests in subjects with pairwise information.

**Additional file 3:** **Table S3.** Comparison between combination of tests and single tests and between the different combinations of tests.

## Data Availability

The datasets generated during and/or analysed during the current study are not publicly available but are available from the corresponding author on reasonable request.

## Abbreviations

CIs: Confidence intervals
CLIA: Chemiluminescent immunoassays
ELFA: Enzyme-linked fluorescent assay
ELISA: Enzyme- linked immunosorbent assays
EUA: Emergency Use Authorizations
FDA: US Food and Drug Administration
FIND: Foundation for Innovative New Diagnostics
IQRs: Median and interquartile ranges
IRR: Incidence rate ratios
LFIA: Rapid lateral flow immunoassays
MIS-C: Multisystem Inflammatory Syndrome in Children
POC: Point-of-care
rRT-PCR: Real-time reverse-transcriptase polymerase chain reaction
SARS-CoV-2: Severe Acute Respiratory Syndrome Coronavirus 2
SDs: Standard deviations
WHO: World Health Organization
