# Supplementary material for: Sensitivity of three commercial tests for SARS-CoV-2 serology in children: an Italian multicentre prospective study
Source: Ital J Pediatr. 2022 Dec 2;48:192. doi: 10.1186/s13052-022-01381-9 (PMC9716520; doi:10.1186/s13052-022-01381-9)
Supplement: Supplementary file 2 — Additional file 2: Table S2. Direct comparison of sensitivity and 95%CI between tests in subjects with pairwise information. [file 13052_2022_1381_MOESM2_ESM.doc]

*Table S2.* Direct comparison of sensitivity and 95%CI between tests in subjects with pairwise information.

|  | **0-14 days** | | | **15-28 days** | | | **29-84 days** | | |
| --- | --- | --- | --- | --- | --- | --- | --- | --- | --- |
| **Test** | **Positive, N** | **Sensitivity (95%CI)** | **p-value** | **Positive,**  **N** | **Sensitivity (95%CI)** | **p-value** | **Positive, N** | **Sensitivity (95%CI)** | **p-value** |
| **Diesse IgG**  **vs**  **Roche S** | 14  vs  32 | 0.23 (0.13-0.35)  vs  0.52 (0.39-0.65) | **< 0.001** | 4  vs  14 | 0.29 (0.08-0.58)  vs  1 (0.77-1) | NA | 1  vs  15 | 0.05 (0.00-0.26)  vs  0.79 (0.54-0.94) | **< 0.001** |
| **Diesse IgG**  **vs**  **Roche N** | 13  vs  27 | 0.25 (0.14-0.40)  vs  0.53 (0.38-0.67) | **0.001** | 3  vs  10 | 0.27 (0.06-0.61)  vs  0.91 (0.59-1) | 0.02 | 1  vs  17 | 0.05 (0.00-0.24)  vs  0.81 (0.58-0.95) | **< 0.001** |
| **Roche S**  **vs**  **Roche N** | 37  vs  34 | 0.57 (0.44-0.69)  vs  0.52 (0.04-0.65) | 0.45 | 14  vs  12 | 1 (0.77-1)  vs  0.86 (0.57-0.98) | NA | 17  vs  17 | 0.85 (0.62-0.97)  vs  0.85 (0.62-0.97) | NA |
