# Supplementary material for: Sensitivity of three commercial tests for SARS-CoV-2 serology in children: an Italian multicentre prospective study
Source: Ital J Pediatr. 2022 Dec 2;48:192. doi: 10.1186/s13052-022-01381-9 (PMC9716520; doi:10.1186/s13052-022-01381-9)
Supplement: Supplementary file 3 — Additional file 3: Table S3. Comparison between combination of tests and single tests and between the different combinations of tests. [file 13052_2022_1381_MOESM3_ESM.doc]

*Table S3.* Comparison between combination of tests and single tests and between the different combinations of tests.

|  | **0-14 days** | **15-28 days** | **29-84 days** |
| --- | --- | --- | --- |
| **Tests** | **P** | **P** | **P** |
| Diesse + Roche S *vs* Roche S | 0.40 | 1 | 1 |
| Diesse + Roche S *vs* Diesse | **<0.001** | **<0.001** | **<0.001** |
| Diesse + Roche N *vs* Roche N | 0.33 | 0.71 | 1 |
| Diesse + Roche N *vs* Diesse | **<0.001** | **0.003** | **<0.001** |
| Roche S + Roche N *vs* Roche S | 0.27 | 1 | 0.23 |
| Roche S + Roche N *vs* Roche N | 0.12 | 0.09 | 1 |
| Diesse + Roche S *vs* Diesse + Roche N | 0.78 | 0.11 | 0.23 |
| Diesse + Roche N *vs* Roche S + Roche N | 0.58 | 0.34 | 1 |
